# ChatGPT as a New Tool to Select a Biological for Chronic Rhino Sinusitis with Polyps, “Caution Advised” or “Distant Reality”?

**DOI:** 10.3390/jpm14060563

**Published:** 2024-05-24

**Authors:** Federico Sireci, Francesco Lorusso, Angelo Immordino, Manuela Centineo, Ignazio Gerardi, Gaetano Patti, Simona Rusignuolo, Riccardo Manzella, Salvatore Gallina, Francesco Dispenza

**Affiliations:** 1 Otorhinolaryngology Section, Department of Precision Medicine in Medical, Surgical and Critical Care (Me.Pre.C.C), University of Palermo, Via del Vespro 129, 133, 90127 Palermo, Italy; federico.sireci@unipa.it; 2 Otorhinolaryngology Section, Biomedicine, Neuroscience and Advanced Diagnosics Department (BiND), University of Palermo, Via del Vespro 129, 133, 90127 Palermo, Italy; francesco.lorusso@policlinico.pa.it (F.L.); ignazio.gerardi@community.unipa.it (I.G.); gaetano.patti@community.unipa.it (G.P.); simona.rusignuolo@community.unipa.it (S.R.); riccardo.manzella@community.unipa.it (R.M.); salvatore.gallina@unipa.it (S.G.); francesco.dispenza@unipa.it (F.D.); 3 Digital Consultant Freelance, 90100 Palermo, Italy; m.centineo@hotmail.it

**Keywords:** artificial intelligence, ChatGPT, chronic rhinosinusitis, nasal polyps, biological therapy, monoclonal antibodies

## Abstract

ChatGPT is an advanced language model developed by OpenAI, designed for natural language understanding and generation. It employs deep learning technology to comprehend and generate human-like text, making it versatile for various applications. The aim of this study is to assess the alignment between the Rhinology Board’s indications and ChatGPT’s recommendations for treating patients with chronic rhinosinusitis with nasal polyps (CRSwNP) using biologic therapy. An observational cohort study involving 72 patients was conducted to evaluate various parameters of type 2 inflammation and assess the concordance in therapy choices between ChatGPT and the Rhinology Board. The observed results highlight the potential of Chat-GPT in guiding optimal biological therapy selection, with a concordance percentage = 68% and a Kappa coefficient = 0.69 (CI95% [0.50; 0.75]). In particular, the concordance was, respectively, 79.6% for dupilumab, 20% for mepolizumab, and 0% for omalizumab. This research represents a significant advancement in managing CRSwNP, addressing a condition lacking robust biomarkers. It provides valuable insights into the potential of AI, specifically ChatGPT, to assist otolaryngologists in determining the optimal biological therapy for personalized patient care. Our results demonstrate the need to implement the use of this tool to effectively aid clinicians.

## 1. Introduction

Artificial Intelligence (AI) encompasses the development of computer systems capable of performing tasks traditionally requiring human intelligence, such as learning from experience, reasoning, problem-solving, understanding natural language, and visual perception [1]. A notable AI model gaining global recognition is the Chat-Generative Pre-Trained Transformer (ChatGPT), which boasts over 175 billion parameters. Released by OpenAI in November 2022, this chatbot extracts information from diverse online sources, refining its text generation through human feedback [2]. Chat-GPT learns linguistic models from extensive text data, making it adept at comprehending and responding to queries in various contexts.

One promising application of ChatGPT lies in healthcare, potentially enhancing patient–doctor interactions and streamlining medical processes. It could serve as a virtual assistant, answering patient queries, scheduling appointments, and providing information on diagnoses and treatments. In specific medical specialties, ChatGPT could aid in explaining complex conditions, support telehealth services, and assist with medical education [3]. Its natural language processing proficiency could enable efficient data retrieval, aiding in remote patient monitoring and administrative tasks. While offering potential in diagnostic support, it could also play a role in personalized patient education and emotional support. The diverse applications of ChatGPT could contribute to improved accessibility, communication, and efficiency within the healthcare sector [4]. 

In cardiology, ChatGPT aids healthcare providers in interpreting intricate diagnostic results. It contributes to a better understanding of test outcomes, assists in patient counseling, and offers lifestyle recommendations for cardiovascular health. Additionally, the model proves valuable in the remote monitoring and management of chronic cardiac conditions [5]. Oncology witnesses ChatGPT as a vital tool for disseminating information on various cancer types, treatment options, and potential side effects. Beyond its informative role, the model serves as a source of emotional support for patients and their families, addressing concerns related to cancer diagnosis, treatment plans, and survivorship [6]. In neurology, ChatGPT acts as an educational guide, simplifying complex concepts, explaining diagnostic procedures, and providing information on available treatment options. Moreover, it aids in raising awareness about neurological disorders and contributes to destigmatizing mental health issues [7]. Pediatrics benefits from ChatGPT as an educational resource for parents, offering guidance on child development, vaccination schedules, and common pediatric illnesses. By answering parental queries and promoting proactive healthcare practices, ChatGPT supports parents in making informed decisions for the well-being of their children [8]. Orthopedics sees the application of ChatGPT in providing information on musculoskeletal conditions, explaining surgical procedures, and offering postoperative care instructions. This assists both patients and orthopedic professionals in ensuring better understanding and adherence to treatment plans [9]. Dermatology leverages ChatGPT to educate patients about skin conditions, skincare routines, and potential treatment options. The model aids in recognizing common skin issues, offering initial guidance and advising on when to seek professional dermatological consultation [8]. In psychiatry, ChatGPT contributes to destigmatizing mental health issues by providing accurate information on various psychiatric conditions. It offers coping strategies, encourages open conversations about mental health, and assists individuals in understanding the importance of seeking professional help when needed [10].

With the advent of this useful tool, numerous studies have been conducted regarding its use. However, studies regarding its application in otolaryngology are still limited.

Chronic rhinosinusitis (CRS) in adults is a persistent inflammatory condition affecting the nasal mucosa and paranasal sinuses, manifesting in symptoms such as nasal blockage, congestion, discharge, facial pain, and reduced smell lasting 12 weeks or more. Its prevalence exceeds 10% in European and U.S. adults, with chronic rhinosinusitis with nasal polyps (CRSwNP) accounting for 5% and causing significant morbidity and diminished health-related quality of life. CRS is categorized into two phenotypes: CRS without nasal polyps (CRSsNP) and CRSwNP, each exhibiting distinct symptomatology and inflammation. The inflammatory pathways classify CRS into type 2 and non-type 2 endotypes. CRSwNP predominantly demonstrates type 2 inflammation, involving innate and adaptive immune systems, marked by high levels of ILC2s, Th2 cells, and cytokines like IL-4, IL-5, and IL-13, with tissue eosinophilia and elevated IgE levels. On the other hand, non-type 2 CRS is linked to Th1/Th17-mediated immune responses, featuring cytokines such as IL-17A, IL-8, IFN-γ, and neutrophilic inflammation. Type 2 inflammation, associated with comorbidities like asthma, leads to increased disease severity and morbidity compared to non-type 2 inflammation, necessitating more surgeries and extensive medical interventions for patients with type 2 inflammation.

In medical or surgical therapy or a combination, complications, uncontrolled symptoms, or recurrence may arise. 

To address uncontrolled CRS with type 2 inflammation, new biologicals, like monoclonal antibodies, are available. However, a lack of tests to evaluate molecular biomarkers hinders personalized medicine for CRS patients. Prescription criteria for biological target therapy in CRSwNP rely mainly on clinical and histological/blood test results [11,12,13].

This study aims to assess if the chosen biological therapy for our CRS patients aligns with recommendations that ChatGPT (version 4.0) would propose in similar cases, addressing the need for improved diagnostic markers or technologies.

## 2. Materials and Methods

### 2.1. Study Sample

An observational cohort study was conducted involving patients evaluated by four otolaryngologists specializing in rhinology and four resident physicians (Rhinology Board) at the Otorhinolaryngology Section of the University Hospital “Policlinico Paolo Giaccone” in Palermo. This study aimed to determine the most suitable biological therapy, mutually agreed upon from options like dupilumab, mepolizumab, and omalizumab, prescribed following the criteria validated by the Italian Medicines Agency (AIFA) for CRSwNP treatment. Data on medical history were collected by assessing age, gender, associated comorbidities, and concurrent conditions such as atopic dermatitis and asthma, allergic history, allergy to non-steroidal anti-inflammatory drugs (NSAIDs), intranasal corticosteroid (INC) use, systemic corticosteroid therapy (SCS), history of endoscopic sinus surgery, the number of previous surgeries, immunoglobulin E (IgE) levels (kU/L), blood eosinophils (cells per mm^3^), sinonasal outcome test 22 (SNOT-22), nasal polyp score (NPS), and the “Sniffin’ Stick” test. The inclusion criteria were those utilized by the Italian Medicines Agency (AIFA) for prescribing biological drugs in Italy: patients suffering from severe CRSwNP (NPS ≥ 5 and/or SNOT-22 ≥ 50); failure of previous medical therapy with corticosteroids (side effects or lack of efficacy) and/or previous endoscopic sinus surgery (complications or lack of efficacy); and absence of concomitant therapy with biological drugs. Patients with eosinophilic granulomatosis with polyangiitis (EGPA) and those who were pregnant or under 18 years of age were excluded from this study. This study did not require ethical approval as no patient-level data were used. All aspects of the study were conducted in strict accordance with the Declarations of Helsinki.

### 2.2. Tools Used in Evalutaions of Patients

For the subjective assessment, the Italian version of the sinonasal outcome Test-22 (I-SNOT-22) was utilized. This version is widely employed in clinical practice due to its simplicity, intuitiveness, and the short time required for completion. The questionnaire comprises 22 items associated with CRS, each scored from 0 to 5, resulting in a total score range of 0–110 (higher scores indicating more severe symptoms). It evaluates the intensity of complaints that patients have experienced in the past weeks due to CRS. The SNOT-22 items are categorized into two groups: questions concerning physical symptoms (items 1–12), covering rhinologic, ear, and facial symptoms, and questions related to health; and quality of life (items 13–22), encompassing sleep function and psychological issues [14,15].

The nasal polyp score (NPS) serves as a clinician-reported gauge, evaluated post-endoscopic examination of nasal cavities. Scoring ranges from 0 to 4 per nostril, where 0 signifies an absence of visible nasal polyps and 4 denotes the complete blockage of the nasal cavity due to nasal polyps. The cumulative scores for both left and right sides provide a potential total score spanning 0–8, where elevated scores signify more substantial nasal polyps and heightened disease severity [16].

The identification test is an integral component of the “Sniffin’ Sticks” 16 items identification test (SS-I) (Burghart instruments, Wedel, Germany), a standardized assessment for evaluating olfactory dysfunction. Based on 16 prevalent odors, each is presented through a forced multiple-choice format with a list of four items (three distractors and one target). An intact sense of smell is established when the patient achieves a score of ≥12 correct answers out of 16. Patients scoring between 9 and 11 are categorized as hyposmic, while those with scores ≤ 8 are designated as anosmic [17].

**Figure 1:**
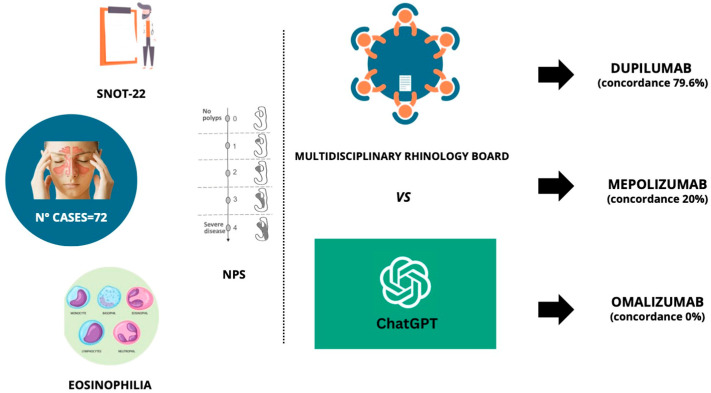
Study protocol summary. SNOT-22 = sinonasal outcome test 22; NPS = Nasal polyps score.

Concerning the dialogue with ChatGPT, the parameters were evaluated using ChatGPT (version 4). A separate chat session was employed for each case, presenting all the collected information. The questionnaire was modeled after a real-life TB panel discussion format. The question posed to ChatGPT was “What is the best biological treatment between Dupilumab, Mepolizumab, and Omalizumab in this patient?”. No patient identification information was provided to ChatGPT. The answers from ChatGPT were collected. The same clinical case information was provided to the Rhinology Board, which, after comparison, offered its opinion on the most appropriate therapeutic strategy. The Rhinology Board’s recommendations were accepted for treatment in all the patients. Subsequently, the level of agreement between the Rhinology Board and ChatGPT regarding the choice of biological therapy was evaluated (Figure 1). 

### 2.3. Statistical Analysis

Quantitative and continuous variables were presented as the mean ± standard deviation (SD). The Pearson Chi-square test was utilized for qualitative variables, and the Student *t*-test was employed to assess the difference between the means of quantitative variables. Statistical significance was considered for *p*-values < 0.05. The Kappa correlation coefficient (k) was used to analyze the agreement between experts in rhinology and ChatGPT 4.0, with the following interpretation guidelines: k < 0.4: poor correlation; k [0.4–0.75]: intermediate correlation; k > 0.75: good correlation. All statistical analyses were conducted using free and validated online tools (http://justusrandolph.net/kappa/; and https://biostatgv.sentiweb.fr/; accessed on 30 November 2023).

## 3. Results

According to our inclusions and exclusions criteria, a total of 72/100 patients affected by CRSwNP were enrolled and in treatment with biological therapy. 

Among them, 45 were men (62.5%), while 27 were women (37.5%), with a mean age of 57.4 (SD + 13.67) years. Table 1 summarize the qualitative parameters of the patients. 

**Figure 2:**
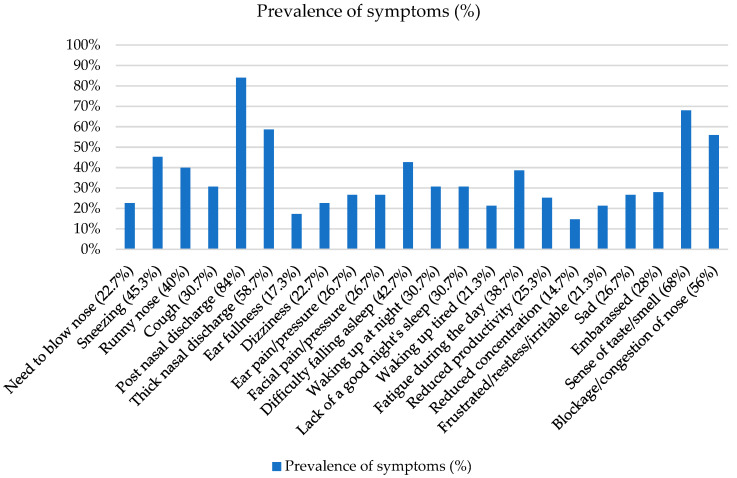
Prevalence of symptoms (%).

Regarding the presence of typical comorbidities of Type 2 inflammation, 44 patients (61.1%) had a history of allergic conditions, 15 (20.8%) had a history of atopic dermatitis, 18 (25%) had a history of NSAID use, and 46 (63.9%) had asthma. Among all patients, 83.4% were undergoing treatment with intranasal corticosteroids. Thirty-eight patients (52.8%) were being treated with oral corticosteroids and were unresponsive or non-compliant. It was noted that 18 (25%) patients were intolerant to non-steroidal anti-inflammatory drugs (NSAIDs). Fifty-seven patients (79.2%) had undergone endoscopic sinus surgery (ESS) previously. The mean value of the sinonasal outcome test 22 (SNOT-22) before treatment was 57.7 (SD + 19.4), with a mean nasal polyps score (NPS) before treatment of 6.5 (SD + 1.2), and a mean sniffin’ stick test score of 3.2 (+3.7). In the blood tests, eosinophils and IgE were 460.4 (+238.9) cells per mm3 and 187.7 (+240.3) kU/L, respectively. In medical history, the main symptoms reported by patients were postnasal discharge (84%), altered sense of taste/smell (68%), thick nasal discharge (58.7%), and blocked/congested nose (56%). Figure 2 reports the prevalence of symptoms.

Table 2 shows the various biological therapies selected by ChatGPT and the Rhinology Board. ChatGPT and experts shared a common answer in 68% of cases, with a Kappa coefficient of 0.69 (CI95% [0.50; 0.75]). The Student *t*-test and Pearson chi-square tests did not reveal statistically significant differences when assessing the role of each parameter in the final therapy decisions made by the Rhinology Board and ChatGPT (*p* > 0.05).

Over 72 patients, 4 with concordance on dupilumab experienced side effects (2 psoriasiform dermatitis and 2 eosinophilia > 2500 × mm^3^) and opted not to pursue further biological therapy. Among the 23 (31.9%) patients with discordance, 3 were prescribed omalizumab, with a noted benefit in 2 patients, while the other switched to dupilumab, with a noted benefit (reduced NPS and Snot-22). The characteristics of these responsive patients were the presence of a high level of IgE (>300 kU/L) along with concomitant inhalant allergy. In six patients prescribed mepolizumab despite Chat-GPT’s recommendation for dupilumab, they benefited from mepolizumab at 6 months, so no switch was made. For 14 patients advised to take dupilumab by the Rhinology Board but mepolizumab by Chat-GPT, no switch occurred at 6 months due to the beneficial effects of the prescribed biologic therapy. Therefore, all the patients were treated according to the Rhinology Board’s recommendation, and none of their decisions over follow-up were overturned by ChatGPT’s decision.

## 4. Discussion

CRS encompasses a range of conditions characterized by distinct clinical presentations and pathogenic mechanisms. Traditionally, CRS has been clinically dichotomized into CRSsNP) and CRSwNP, assuming a predominant role of T-helper 1 cells in the former and T-helper 2 cells in the latter. However, ongoing research has revealed a more intricate immunologic profile, indicating overlap and coexistence of endotypes within the same patient. Non-eosinophilic inflammation, dominated by Th1/Th17 pathways, may be associated with CRSwNP, while CRSsNP patients may express a Type 2 cytokine profile.

Considering comprehensive endotyping studies providing insights into the underlying cellular and molecular inflammatory mechanisms associated with CRS, the EPOS 2020 group has opted for a paradigm shift in CRS management. Recognizing the need to move away from phenotype-based classification (CRSsNP vs. CRSwNP), the focus is now on a new classification based on the localization of the disease, distinguishing between unilateral and diffuse (always bilateral) presentations. Further stratification is based on endotypes, categorizing them as type 2 or non-type 2 diseases. In cases of multiple coexisting endotypes in the same patient, the authors suggest identifying the dominant one to establish an optimal personalized therapeutic approach.

Approximately 80% of diffuse CRS cases in Western countries exhibit a dominant Type 2 response, primarily driven by key Type 2 cytokines (IL4, IL5, IL13, etc.) and circulating/local IgE, with eosinophilia as a characteristic signature. Presently, both allergic (IgE-mediated) and non-allergic pathways are recognized in the pathophysiology of underlying eosinophilia, representing the ideal immune profile for severe CRSwNP candidates for biologics. Recent position papers recommend confirming Type 2 inflammation in these patients through systemic eosinophil and IgE counts. The intensity of local eosinophilic infiltration and the overall inflammatory response correlate closely with prognosis and disease severity, emphasizing the need for institutional protocols in sampling, storing, and processing sino-nasal mucosa samples.

Various techniques, including nasal biopsy, nasal brushing or scraping (nasal cytology), nasal lavage fluid, and nasal suctioning of secretions, are used to define local inflammation. Authors suggest quantifying eosinophils per high-powered field (hpf), with the EPOS steering group specifying a cutoff of eosinophils >10/hpf to confirm Type 2 inflammation. Cut-offs for other procedures are yet to be established, necessitating specific studies. Blood eosinophilia (>250/microliter) and total IgE levels (>100 kU/L) serve as specific cutoffs for identifying Type 2 disease, with periostin and other potential biomarkers under investigation.

The combination of phenotyping (responsiveness to various treatments) and endotyping (blood/local eosinophils or neutrophils, TH-cell populations, cytokine levels, IgE, periostin, and other biomarkers) currently offers the best means by which to predict the natural course of disease and prognosis after surgery. Authors aim to identify optimal predictive methods to guide counseling on expected surgical outcomes and postoperative medical management for effective symptom control. Ultimately, recognizing endotypes is crucial for tailoring individualized therapy [18].

The advent of biological drugs in chronic rhinosinusitis with polyps has shown encouraging results in its treatment. Currently, the approved biological therapies for CRSwNP are dupilumab, mepolizumab, and omalizumab all, with subcutaneous administration [12]. These therapies have different mechanisms of action and different collateral effects. Dupilumab is a fully human monoclonal antibody targeting the α-chain subunit of IL-4 receptors (Type 1 and type 2 IL-4Rα) and inhibiting IL-4/IL-13 signaling. The recommended dose is 300 mg every 2 weeks by a device auto-injector. Home administration is allowed. This mechanism can cause side effects such as injection site reactions, conjunctivitis, and transitory eosinophilia (<2% of cases) [19,20,21,22]. Omalizumab is the longest-lived monoclonal antibody approved since 2003 for the treatment of moderate to severe persistent allergic asthma in more than 90 countries [13]. It was designed to treat IgE-mediated disease by reducing the concentration of free IgE in blood and tissue. The injection in this case is every 2–4 weeks, and dosing and frequency level are determined by serum total IgE level and body weight. This therapy can be associated with headache, dizziness, arthralgia, abdominal pain upper, and injection site reactions [23]. Mepolizumab is a monoclonal antibody binding to IL-5, a crucial cytokine in the activation and maturation of eosinophils. The administration is 100 mg monthly subcutaneous injections regardless of weight. In this case, the patient can develop nasopharyngitis, headache, and injection site reaction [24,25].

In Italy, these therapies were approved by the Italian Agency of Drugs (AIFA) in 2020 for dupilumab, 2022 for omalizumab, and 2023 for mepolizumab for adult patients with severe CRSwNP (assessed by an NPS score ≥ 5 or a SNOT-22 score ≥ 50) for whom therapy with SCS and/or surgery does not provide adequate disease control, in addition to background therapy with INC.

These criteria were partially inferred from the EPOS 2020 guidelines [12,13], which, for the first time, proposed five criteria with which to select CRSwNP patients eligible for biologics: evidence of type 2 disease (tissue eosinophils ≥ 10/hpf or blood eosinophils ≥ 250/microliter or total IgE ≥ 100); the need for at least two courses of SCS per year or long-term (>3 months) low-dose steroids or contraindication to systemic steroids; significantly impaired quality of life (SNOT-22 ≥ 40); anosmia on smell test and/or comorbid asthma requiring regular inhaled corticosteroid.

Most of these criteria are clinical and do not resolve the problem of patient stratification to choose the appropriate biological therapy for each case and the problem of cost. Indeed, the selection of the most suitable biological therapy could potentially lead to cost reduction, a matter that remains contentious for several reasons. Certain studies, as highlighted by specific authors, have shown that biologics tend to be cost-efficient, particularly in patients whose conditions are inadequately controlled with standard care. However, the debate persists, with various investigations emphasizing that the cost-effectiveness of biologics could be further optimized through actions such as pharmaceutical companies lowering prices. Additionally, proponents of this view suggest that clinicians should focus more on subgroups, such as clear responders and individuals requiring more frequent prescriptions of systemic corticosteroids (SCS), to better justify the costs associated with biologic therapies [26,27]. 

In this scenario, AI and ChatGPT can represent a possible new tool in the decision-making process for biological therapy.

One of the notable features of the ChatGPT algorithm is its capacity to generate responses that mimic human-like patterns across a diverse array of questions and prompts. This proficiency is a result of the algorithm undergoing training on an extensive textual database, enabling it to grasp the intricacies of language and produce responses that are both contextually pertinent and grammatically accurate.

The results of our study, for the first time, demonstrate an intermediate global degree of consensus between ChatGPT and the Rhinology Board of our hospital (49/72 patients with a concordance percentage of 68%). In general, ChatGPT supports its answers as follows: Dupilumab is often the primary choice for CRSwNP because, according to the literature, it is most effective in reducing polyp size and addressing anosmia, especially in cases with coexisting atopic dermatitis and asthma. The choice of Dupilumab is more frequent and exhibits greater concordance because it effectively targets the upstream cascade of type 2 inflammation, rendering it the drug with the highest predictive efficacy. Omalizumab is recommended when CRSwNP patients are allergic with a high level of IgE, with a mean of 289.5 (SD + 359.6) in patients chosen by ChatGPT, compared to 295.6 (SD + 49.2) in cases chosen by our board, without a statistically significant difference (t = 0.028; *p* = 0.9). Mepolizumab is recommended for treating CRSwNP when accompanied by asthma (chi-square = 0.0857; *p*-value = 0.769698) and a high level of eosinophilia, with an average of 452 (SD + 275.1) in patients selected by ChatGPT compared to 674 (SD + 253.5) in cases chosen by our Rhinology Board (t = 0.028; *p* = 0.9). Thus, while the absence of concordance between ChatGPT and the Rhinology Board in recommending omalizumab and Nucala precludes definitive conclusions, it underscores the imperative for further investigation into patient-specific profiles to refine treatment strategies and enhance clinical decision-making.

It is important to note that in the case of asthma, the difference is not statistically significant, unlike eosinophilia [28]. This suggests that for our Rhinology Board, the perceived effectiveness of the drug is higher only in the presence of very high eosinophilia values, indicating that AI models operate based on data patterns with a potential absence of clinical intuition.

Our study underscores the potential of AI, specifically Chat-GPT, to assist otolaryngologists in determining the optimal biological therapy for patients with CRSwNP. Chat-GPT demonstrated a substantial level of agreement with the participating otolaryngologists. This research marks a noteworthy stride in enhancing the management of a pathology that currently lacks robust biomarkers. However, a multicentric study with a large scale of cases could be useful to confirm and validate our preliminary results. [17]

To date, ChatGPT recommendations cannot be taken at face value without specialist verification since it is not uncommon for the chatbot to provide erroneous information.

In fact, the results strongly inform that currently ChatGPT does not have a place in clinical practice.

For omalizumab, the three cases recommended by the board were not recommended by ChatGPT. Three cases recommended for omalizumab by ChatGPT were not recommended by the board, and the concordance was zero. For mepolizumab, out of 12 cases recommended by the board, only 2 of them were also recommended the drug by ChatGPT. Out of the 14 cases in which ChatGPT recommended a biological therapy, only 2 were accepted by the board. That means an unnecessary treatment of 12 subjects and it missing out on 10 cases that actually needed the drug. The results are better for dupilumab. Still, for 12 out of 59 cases recommended by the board, ChatGPT missed, and for 8 cases which ChatGPT recommended, the board did not recommend them. A prominent limitation lies in the potential for the algorithm to produce responses that are biased or inaccurate, particularly if the training data incorporates biases or inaccuracies. Additionally, the algorithm may encounter challenges when dealing with intricate or abstract concepts that demand a deeper understanding of context or cultural subtleties not encompassed in the training data.

Another restriction of the ChatGPT algorithm is its incapacity to genuinely comprehend the meaning or intent behind a question or prompt. Instead, it relies on statistical patterns within the training data to formulate responses, which may not consistently capture the true meaning or intent of the question. This could result in misunderstandings or miscommunications, especially if the user’s question or context is ambiguous or unclear. Overcoming these limitations requires the provision of clear and specific questions or prompts.

An additional limitation specific to ChatGPT4 is its constrained literature search capability, limited to papers up to the year 2021. Furthermore, ChatGPT does not furnish cutoffs or specific values for all criteria; instead, it offers generalized concepts and intriguing decision-making algorithms, anchored in established evidence.

Overcoming such limitations, coupled with a potential enhancement in ChatGPT’s diagnostic yield through the integration of clinical information with its recently acquired image processing capability, could signify a significant advancement. However, this could raise complex ethical concerns regarding data storage and processing. 

Healthcare professionals need to carefully assess how to best implement new resources, ensuring both the safety and feasibility of patient care and supporting our future studies.

## 5. Conclusions

Our study highlights that the use of ChatGPT to aid decision making is a distant reality. The model needs to be trained with large databases to gain acceptable proficiency to aid medical professionals in making appropriate clinical decisions. While showing promise, Chat-GPT’s recommendations require specialist verification due to possible errors and biases. Its limitations include reliance on statistical patterns, restricted literature search, and the need for clearer prompts. Integrating clinical data and image processing could enhance Chat-GPT’s diagnostic ability, but ethical concerns must be addressed. Healthcare professionals should cautiously implement such tools to ensure patient safety and support ongoing research. This means that the tool still needs to evolve, and additional training is needed. Additionally, it is worth noting that the work is based on data from a single center; therefore, a multicenter study could provide further insights. In fact, the small sample size greatly affects the reliability of the results and the agreement rate of analysis. 

## Figures and Tables

**Table 1:** General characteristics of the study population.

	N = 72
Age, yr	57.4 (±13.67)
Sex	M = 45 (62.5%)
	F = 27 (37.5%)
Allergy	44 (61.1%)
NSAIDs allergy	18 (25%)
Atopic Dermatitis	15 (20.8%)
Asthma	46 (63.9%)
INC	60 (83.4%)
SCS	38 (52.8%)
SNOT-22	57.7 (±19.5)
NPS	6.5 (±1.2)
Sniffin’ stick test	3.2 (±3.7)
Eosinophilia	460.4 (±238.9) × mm^3^
IgE	187.7 (+240.3) kU/L
Previous ESS	57 (79.2%)

NSAIDs = non-steroidal anti-inflammatory drugs; INC = intranasal corticosteroid; SCS = systemic corticosteroid; SNOT-22 = sinonasal outcome test 22; NPS = nasal polyps score.

**Table 2:** Comparison of treatment recommendations between Rhinology Board and Chat GPT for different biological therapies.

	Rhinology Board [N (%)]	ChatGPT [N (%)]	%Concordance
Dupilumab	59 (82%)	55 (76.4%)	47/59 (79.6%)
Mepolizumab	10 (13.8%)	14 (19.4%)	2/10 (20%)
Omalizumab	3 (4.2%)	3 (4.2%)	0/3 (0%)
Tot			49/72 (68%)

## Data Availability

No new data were created or analyzed in this study. Data sharing is not applicable to this article.
